# Clinical and genetic analysis of epilepsy with myoclonic-atonic seizures caused by SLC6A1 gene variant

**DOI:** 10.3389/fped.2024.1492062

**Published:** 2025-01-21

**Authors:** Zhen Li, Changming Han, Hongwei Zhao

**Affiliations:** ^1^ Department of Pediatrics, Dongguan People’s Hospital (Xiegang), Dongguan, Guangdong, China; ^2^ Department of Pediatrics, Anyang Maternal and Child Health Hospital and Anyang Children’s Hospital, Anyang, Henan, China

**Keywords:** epilepsy with myoclonic-atonic seizures, SLC6A1 gene, developmental delay, levetiracetam, genetic variation

## Abstract

**Objective:**

This research intends to examine the clinical characteristics and genetic diversity of a child experiencing epilepsy with myoclonic-atonic seizures (EMAS) attributed to a variant in the SLC6A1 gene.

**Methods:**

A male child diagnosed with EMAS underwent clinical and electroencephalographic evaluation. Peripheral blood samples were collected for DNA extraction and subsequent whole-exon gene sequencing. For previously identified patients, high-throughput sequencing was utilized, whereas Sanger sequencing was employed for the parents to determine the site of the gene mutation and examine the connection between genotype and phenotype.

**Results:**

The male child showed delays in intellectual and language development before the disease began. At 1 year and 2 months, he had a febrile seizures, which was succeeded by seizures at 2 years and 9 months; these seizures presented as generalized tonic-clonic, myoclonic, and myoclonic-atonic seizures, along with symptoms showing inattention and hyperactivity. After receiving treatment with levetiracetam (50 mg·kg·d^−1^), the child has been free of seizures for the last 8 months. Genetic analysis indicated a heterozygous missense variant of c.263T > C (p.L88P) in the SLC6A1 gene in the child, recognized as a spontaneous mutation that has not been previously documented in the literature.

**Conclusion:**

The variant in the SLC6A1 gene is implicated as one of the etiological factors contributing to EMAS coupled with neurodevelopmental abnormalities. The identification of this novel mutation enriches the spectrum of known SLC6A1 gene variants.

## Introduction

Epilepsy with myoclonic–atonic seizures (EMAS), commonly referred to as Doose syndrome, was first introduced by German physician Hermann Doose et al. in 1970 (1). This syndrome is a complex condition in children, characterized by the presence of multiple types of epileptic seizures, and is considered relatively rare in clinical practice (2). Although the exact cause remains unknown, it may be associated with genetic susceptibility. The usual age of onset lies between 7 months to 6 years, with the highest occurrence noted between 1 and 5 years, alongside a male-to-female ratio close to 2:1. The onset of EMAS is sudden, frequently characterized by a generalized tonic-clonic seizure, followed by the emergence of various forms of generalized seizures. The prognosis for this condition varies significantly among patients. Approximately 70% of individuals respond positively to anti-seizure medications, leading to eventual seizure relief and a favorable prognosis. However, a minority of patients may later develop tonic attacks during the course of the disease, potentially progressing to Lennox-Gastaut syndrome. EMAS may possess a multifactorial genetic basis, as nearly one-third of the affected individuals have a family history indicating different seizure types (3). The clinical manifestations of EMAS are varied, and the prognosis can differ significantly, ranging from mild cases to severe forms such as epileptic encephalopathy. Therefore, timely diagnosis and appropriate treatment may considerably influence the long-term outcomes for those with EMAS. In this case report, we outline the clinical and genetic traits of a child who has a spontaneous mutation in the SLC6A1 gene linked to EMAS. We aim for this report to raise awareness among pediatricians about EMAS, and support early diagnosis and intervention.

## Information and methods

### General information

Clinical information was gathered from a 2-year and 9-month-old male child who visited the hospital in April 2021, presenting a case of “15 days of intermittent fever and 6 seizures in one day.” The child (G3P2) was delivered at full term through spontaneous vaginal delivery, weighing 3,300 g at birth. His birth history showed no significant issues, and his parents were not related by blood. The father experienced febrile seizures during his early years, whereas the mother and the child's 11-year-old sister had no history of seizures. All family members exhibited normal growth and developmental progress. To investigate the underlying cause of the illness, medical exome sequencing was performed following the approval of the hospital's Medical Ethics Committee and with the informed consent of the child's guardians. It was not appropriate or possible to involve patients or the public in the design, or conduct, or reporting, or dissemination plans of our research.

### Exome capture sequencing

Venous peripheral blood (2 ml) that had been anticoagulated with EDTA was obtained from the child and their parents to isolate 3–5 µg of genomic DNA, adhering to the protocols set forth by the Blood DNA Mini kit (Simgen, China). This isolated DNA was subsequently utilized for amplification aimed at creating a panel of genes linked to myoclonic-atonic and psychomotor delay, including SLC6A1, PCDH19, SETD1A, SLC2A1, SLC25A10, GOT2, TBC1D24, KCNQ5, PIGU, RTTN, SCN1A, SCN1B, SCN2A, CHD2, SYNGAP1, and STX1B, among others. A liquid phase capture kit was employed to capture the target genes, which were then analyzed using high-throughput sequencing on the Illumina HiSeq 2000 (Illumina Inc., USA). The sequencing depth averaged a minimum of 200×, and the resulting data underwent bioinformatics analysis of gene sequences to pinpoint potential causative genes.

### Sanger sequencing verification

Primers were crafted for the proposed variant loci, and PCR amplification was performed to confirm the variant, particularly for detecting short fragment deletions or insertions at the identified locus and to describe the variant at that location. The validation primer details for the previous witness and parents are as follows: chromosome position chr3:11059553, forward primer sequence CATATGGGCTTTCCTTGGGC, and reverse primer sequence AACATAGGAGCCAGCTTCCA.

### Analysis of sequencing results

The range of annotation for the raw data from second-generation sequencing includes variants found in each exon, as well as the regions upstream and downstream, extending 10 base pairs in either direction. The identified types of variants consist of missense, synonymous, nonsense, shifted code, whole code, and splice variants, among others. To ensure data integrity, quality control measures categorized variant data with a sequencing coverage depth of less than 20× as low quality. Databases such as dbSNP, ESP6500, ExAC, and other population databases were employed for the classification of single nucleotide polymorphisms and variants of low frequency that are benign. Various prediction software tools were used to assess the conservativeness, pathogenicity, and potential impact of the variants. In addition, databases like HGMD, PubMed, and ClinVar were referenced to gather relevant literature, and the pathogenic nature of the variants was evaluated according to the guidelines set forth by the American College of Medical Genetics and Genomics (ACMG).

## Results

### Clinical data

**Figure 1:**
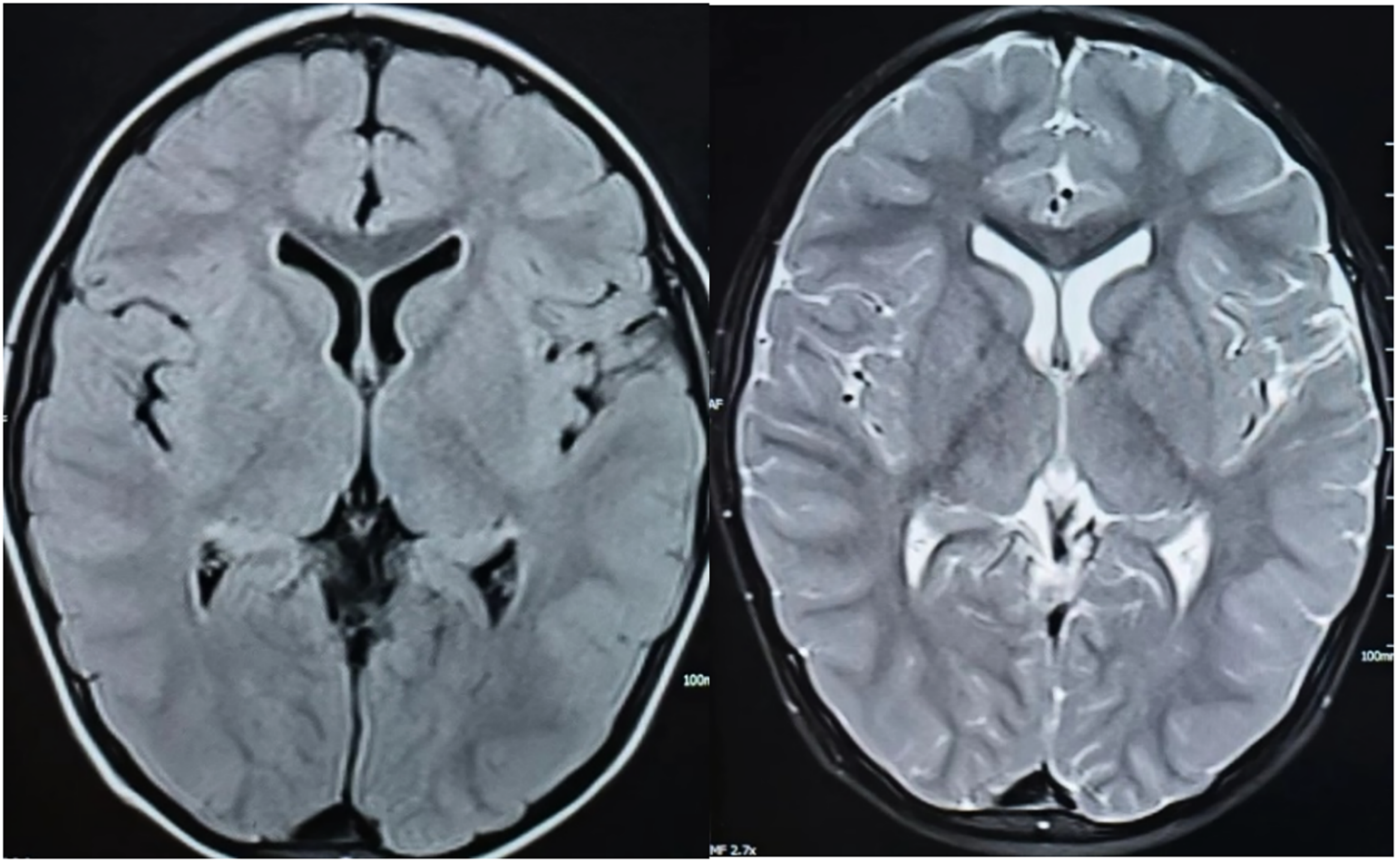
T1 (left) and T2 (right) weighted images of the child's cranial MRI scanning transverse position.

A 2-year and 9-month-old male child had a febrile seizure at the age of 1 year and 2 months, which presented as a generalized tonic-clonic seizure that lasted about 2 min, during which his temperature was recorded at 38.2°C. Cranial computed tomography (CT) and electroencephalography (EEG) performed at a hospital in Anyang City revealed no abnormalities. After reaching 2.5 years of age, the patient experienced several falls while playing in a standing position; however, he was able to rise independently within 3–4 s (a video of the child's seizure was provided by his family). In April 2021, he presented to the hospital following six seizures occurring within one day, some with fever and some without. The first episode involved generalized tonic-clonic seizure (GTCS) associated with a fever reaching 39°C, enduring for about 15 min, which was relieved by an intramuscular dose of midazolam administered. Subsequently, patient had five intermittent seizures, manifesting as myoclonic or myoclonic-dystonic, which occurred with low or no fever, lasting 3–5 s and there has been no fever since admission. Physical examination revealed mild cognitive and language delays, with normal muscle strength and tone. Both deep and shallow reflexes were present, while signs of meningeal irritation and pyramidal tract involvement were absent. The cerebrospinal fluid examination and brain magnetic resonance imaging showed no abnormalities (Figure 1). EEG conducted there was a significant presence of widespread 3–4 Hz low-to-high amplitude slow wave bursts lasting from 2 s or more during both waking and sleeping periods in the interictal phase. The intelligence quotient (IQ) is measured at 24.7 months, while the developmental quotient (DQ) is assessed at 75 months. The patient underwent several convulsive incidents, occurring with and without fever, presenting in several seizure types such as myoclonic atonic and generalized tonic-clonic. The child exhibited delayed language and intellectual development prior to the onset of the disease. Following an increase in the oral Levetiracetam dosage to 50 mg/kg/day, the patient's epileptic seizures were temporarily alleviated. A follow-up EEG revealed extensive slow wave activity with no abnormal discharges detected. Despite undergoing rehabilitation treatment in our hospital for six months, improvement has been minimal.

### Analysis of gene variants

**Figure 2:**
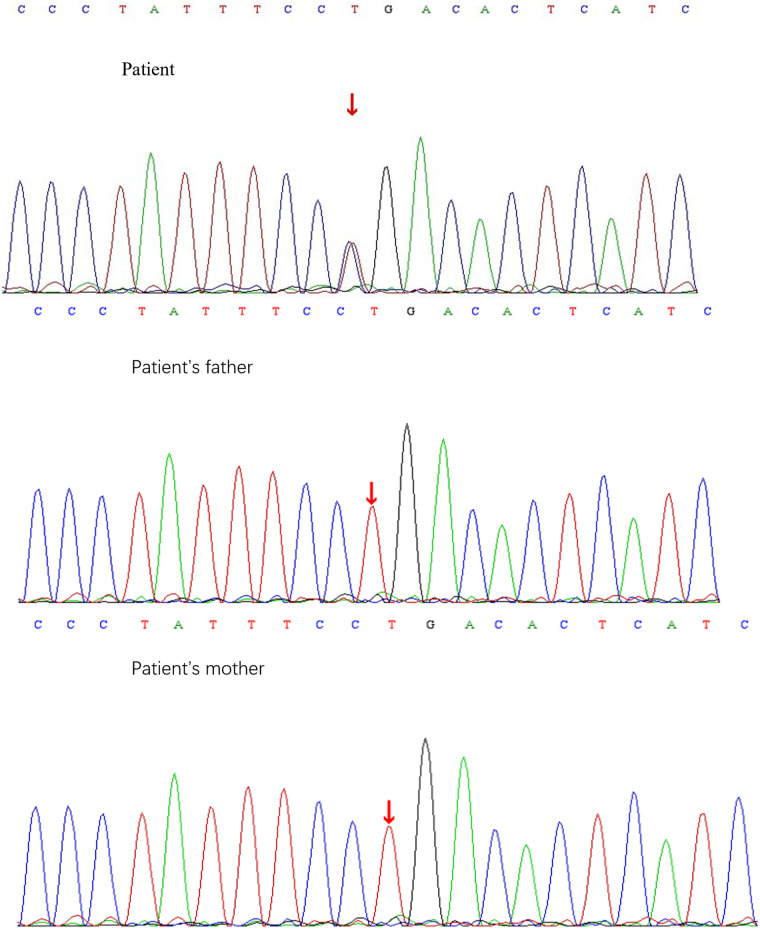
Sanger sequencing results of c.263T > C locus of SLC6A1 gene in children and their parents.

**Figure 3:**
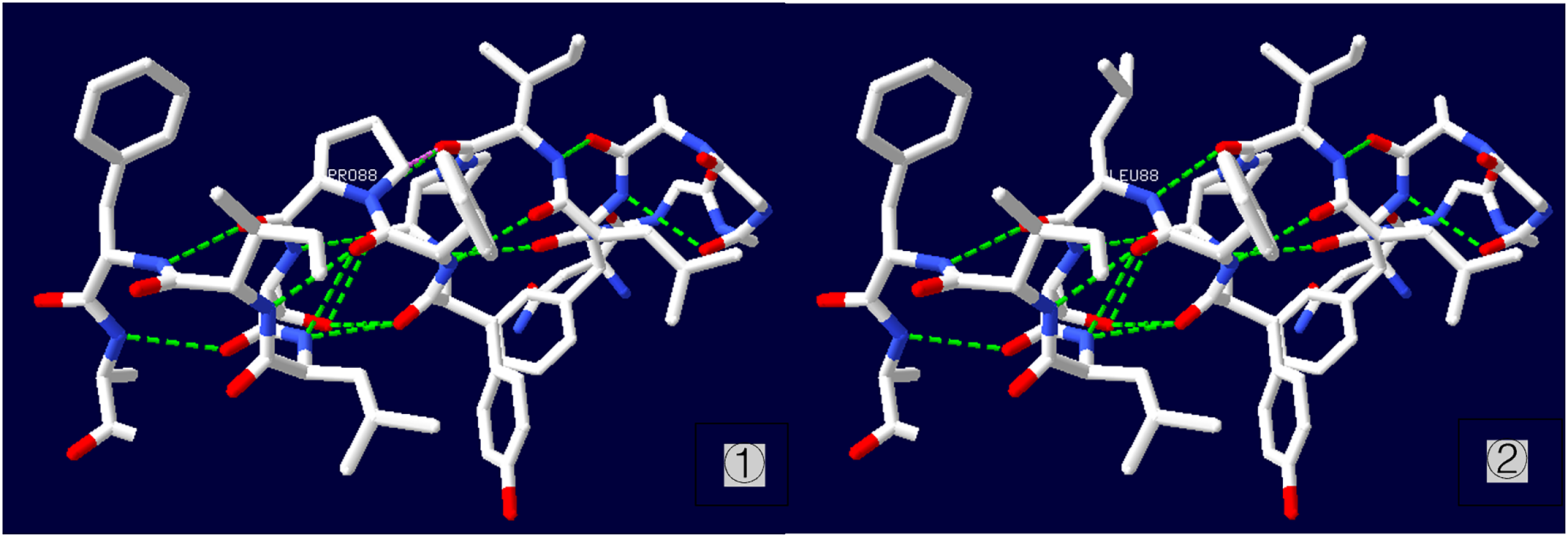
Three-dimensional structure of p.L88P protein ① wild type; ② mutant type.

A heterozygous missense mutation was detected in the SLC6A1 gene (Figure 2), specifically noted as a nucleotide alteration at position 263 from thymine (T) to cytosine (C) (c.263T > C). This change leads to an alteration in the amino acid at position 88, transforming leucine into proline (p.L88P) (Figure 3). Sanger sequencing verified that no variation was present at this location in the parents. This specific variant is categorized as a missense mutation and does not appear in the Thousand Genomes, ESP6500, or dbSNP databases. Moreover, data from the Normal Population database reveals that this variant is of low frequency. Based on ACMG guidelines, the variant is regarded as suspected pathogenic.

## Discussion

The SLC6A1 gene can be found on chromosome 3p25.3 (4) and comprises 16 exons, covering roughly 46.5 kb. This gene exhibits extensive expression in the brains of both humans and various other animal species. *γ*-Aminobutyric acid (GABA) is a vital inhibitory neurotransmitter in the mammalian brain, serving to curb neuronal hyperexcitability through the modulation of inhibitory synaptic transmission (5). The extracellular concentrations of GABA are regulated by four transporters known as GABA transporters (GATs), specifically GAT1, GAT2, GAT3, and BGT1, which are Na/Cl^−^-coupled members of the solute carrier 6 (SLC6) family (6). The SLC6A1 gene encodes the GABA transporter protein GAT1 (7), playing a critical role in the reabsorption of GABA from synapses as well as the removal of GABA from the extracellular environment (8, 9). The primary GABA transporter in the central nervous system is GAT1, which is predominantly found in the brain (10). Alterations in the SLC6A1 gene result in diminished activity of the GABA transporter. Furthermore, the transport of GABA through GAT1 relies on an inward electrochemical gradient and includes the co-transport of Na+ and Cl− across the membrane (5). The functioning of GAT1 involves several steps that depend on the identification of a hydrophobic peptide segment of the newly formed protein by the signal recognition particle (SRP). Following this, the hydrophobic peptide is sent toward the endoplasmic reticulum (ER). Point mutations in SLC6A1 frequently interfere with the typical folding pathway of the transporter, resulting in its retention in a misfolded configuration at the ER level, which negatively impacts GABA transport (11). Research by Crossman et al. (12) revealed that administering the GABA antagonist bicuculline into the lateral chiasma nucleus of monkeys can trigger contralateral limb myoclonic seizures. Currently, the etiology of EMAS remains unknown, although it may be associated with genetic susceptibility. Recent studies have identified mutations in several genes, including SCN1A, SCN2A, SCN1B, STX1B, SLC2A1, GABRG2, CHD2, SYNGAP1, KIAA2022, NEXMIF, and SLC6A1, that can contribute to the phenotype of Doose syndrome (13–15). The identified variants consist of missense variants, nonsense variants, frameshift variants, splice variants, and chromosomal microdeletions, with missense variants being the most frequently observed. Recent studies have indicated a positive correlation between *β*-hydroxybutyrate (*β*-HB), a vital component of ketone bodies (KB), and improved patient outcomes following a stroke. The advantageous effects of *β*-HB are influenced by signaling pathways involving HDAC2/HDAC3 and the GABA transporter 1 (GAT-1), which enhance excitability and phasic GABA inhibition (16). Clinical observations reveal that *β*-HB levels do not strongly correlate with the management of seizures (17), pointing to the SLC6A1 gene as a potential avenue for better understanding the effectiveness of the ketogenic diet in addressing myoclonic-atonic seizures.

In 2015, Johannesen et al. (18) indicated that as many as 4% of individuals with EMAS presented *de novo* variants of the SLC6A1 gene. That same year, Carvill et al. (19) discovered that out of 75 patients with EMAS and SLC6A1 mutations, two were a mother and child sharing comparable clinical symptoms, implying that the variant in SLC6A1 might be particular to EMAS. Our patient showed a heterozygous missense mutation, c.263T > C (p.L88P), in the SLC6A1 gene, identified as a spontaneous mutation and suspected to be pathogenic, which has not been documented before. Carvill et al. (19) recognized SLC6A1 as a contributor to neurodevelopmental disorders through two separate whole-exome sequencing investigations, which uncovered *de novo* mutations in two patients diagnosed with intellectual disability and autism. In 2018, Johannesen et al. (18) analyzed 34 patients who were SLC6A1-positive and noted that most demonstrated language delays and mild to moderate intellectual disability (ID) before the initiation of seizures, although a small number experienced ID without having seizures. Mutations in SLC6A1 were linked to a wide array of phenotypes, which varied from typical development to different levels of language and motor delays, along with epileptic encephalopathy that included seizure types besides myoclonic atonic, with cataplexy and atonic seizures being predominant. In our case, the child exhibited mild intellectual and language development delays prior to the onset of myoclonic-atonic seizures, characterized by inattention, hyperactivity, the ability to communicate with family members but poor language expression, developmental quotient delays, and essentially normal motor development. The child manifested various seizure types, including myoclonic atonic seizures, GTCS, myoclonic seizures, and status epilepticus, without evidence of atonic seizures, which aligns closely with reports from international studies.

In 2019, Angione et al. (20) conducted an analysis involving 77 individuals diagnosed with EMAS. Their findings indicated that 42% of these patients had a familial history of childhood myoclonic-atonic seizures. Moreover, 5% did not have any family background of myoclonic-atonic seizures but had relatives who experienced other neurodevelopmental disorders, such as developmental delays, learning disabilities, and autism. In our case, the father had episodes of febrile seizures during early childhood; nevertheless, his genotypic and phenotypic evaluations were normal. Furthermore, there was no available information about other family members with seizures or neurodevelopmental issues. The variation in clinical phenotypes identified in patients with EMAS and their relatives indicates that the pattern of inheritance could be multifactorial or polygenic. Although the majority of documented cases are widespread, it is broadly recognized that EMAS has a significant genetic basis. Nevertheless, the rate of positive genetic diagnoses remains low, potentially due to the exclusion of some genes currently thought to be associated with EMAS from common myoclonic-atonic gene panels or their recent addition. Further studies are needed to clarify the exact causative genes.

In 2011, Trivisano et al. (21) conducted an analysis of 18 patients diagnosed with EMAS, with 88.9% of them being boys, all of whom exhibited myoclonic dystonic. Among these individuals, 16 achieved remission from seizures within a 42-month period from onset; nonetheless, the length of seizure cessation did not correlate with cognitive outcomes. In over two-thirds of the cases, the initial seizure types identified during the progression of the disease were febrile seizures and febrile GTCS. Our reported case centers on a male child who had a history of febrile seizures before the development of EMAS, with his first seizure being a febrile-induced GTCS. He began experiencing seizures at the age of 2 years and 9 months, displaying both febrile and febrile-absent GTCS as the disease evolved, which is consistent with findings reported internationally. In 2013, Caraballo et al. (22) investigated the electroclinical characteristics of 69 children with EMAS, discovering that almost all patients presented with normal findings in cranial imaging and cerebrospinal fluid tests. Interictal EEG recordings showed widespread peaked, multi-peaked, and wave-like discharges, occurring at a frequency of 2–5 Hz. In 2007, Kilaru et al. (23) noted that nearly all patients with EMAS attained complete seizure remission within a span of 3.5 years, although the outcomes related to cognitive development remained ambiguous. It was also observed that patients with EMAS linked to SLC6A1 gene variants achieved seizure remission after being treated with medications such as valproate, levetiracetam, or zonisamide, while some showed partial improvement when lamotrigine was combined with valproate. In this case, the child's cranial MRI and cerebrospinal fluid examination were normal. The interictal EEG demonstrated widespread slow waves during both wakefulness and sleep, along with a small number of 2 Hz widespread spikes and slow wave paroxysms observed in the sleep phase. Notably, the child remained seizure-free for 10 months following levetiracetam treatment. A subsequent review of the EEG revealed slow background activity, the absence of abnormal discharges, and no signs of cognitive-linguistic regression, which aligns with findings reported in the literature. Unlike many epileptic encephalopathies, the outlook for EMAS shows considerable variability, spanning from mild cases to severe epileptic encephalopathies, as well as from normal cognitive function to profound intellectual disabilities. A favorable prognosis is more probable if seizures cease and the EEG remains free of persistent abnormalities.

The onset of EMAS is rapid, typically beginning with a generalized tonic-clonic seizure, followed by the emergence of various forms of generalized seizures, including myoclonus, myoclonus-atonic, atonic, and atypical absence seizures, often accompanied by frequent seizure activity. Falls may occur as a result of myoclonic, myoclonic-atonic, or atonic seizures, and some children may experience atypical absence status. The electroencephalogram reveals widespread irregular 2.5–3 Hz spinous slow or polyspinal slow complex waves, while synchronized electromyography during myoclonic-atonic or atonic attacks demonstrates a brief electrical rest period. Most patients exhibit normal or near-normal intelligence; however, a minority who are unable to control their seizures in a timely manner may experience cognitive impairments. According to the 2022 ILAE classification, EMAS is categorized as a developmental epileptic encephalopathy (DEE) (24). Mutations in the SLC6A1 gene are predominantly missense mutations. The age of onset is typically early, with a peak occurring between 1 and 3 years of age. The seizure types associated with these mutations include absence seizures, atonic seizures, myoclonic seizures, and myoclonic-atonic seizures, with epileptic seizures being the most prevalent. Prior to seizure onset, there may be indications of psychomotor retardation. While seizures are generally more manageable, affected individuals often experience varying degrees of residual intellectual disability. Additionally, these children are at a higher risk for comorbid neurological conditions, such as autism and attention deficit hyperactivity disorder (25). In this case, the child presented with generalized status epilepticus as the initial symptom. Both the brain MRI and cerebrospinal fluid examination yielded normal results. He had a prior history of febrile seizures, and his electroencephalogram was normal during that period. Family members provided video evidence of the fall attack. Considering the age-related characteristics, history of febrile seizures, family history, presence of multiple seizure types, and developmental delay, the clinical features aligned with those of EMAS. The results from genetic testing further corroborated our assessment.

To sum up, it is advisable to screen for SLC6A1 in children who demonstrate various types of seizures, such as dystonic-atonic seizures, myoclonic seizures, GTCS, and additional seizure variants, especially when there is a history of developmental regression before the disease begins, regardless of the presence of behavioral disorders. Additionally, seizure remission with medication should be considered as part of the evaluation process for early diagnosis and treatment, which may improve the prognosis for these children. This study represents a case analysis with inherent limitations. Consequently, the frequency of every possible genetic cause needs to be evaluated in a substantial multicenter group. In addition, focused assessment of genes that have been previously associated with EMAS would enhance the comprehension of the outcomes related to known genes and aid in discovering new candidate genes.

## Data Availability

The original contributions presented in the study are publicly available. This data can be found here: https://www.ncbi.nlm.nih.gov/bioproject/PRJNA1209639 (accession number: PRJNA1209639).
